# On the Relationship Between the Gini Coefficient and Skewness

**DOI:** 10.1002/ece3.70637

**Published:** 2024-11-28

**Authors:** Meng Lian, Long Chen, Cang Hui, Fuyuan Zhu, Peijian Shi

**Affiliations:** ^1^ Co‐Innovation Centre for Sustainable Forestry in Southern China, State Key Laboratory of Tree Genetics and Breeding, Bamboo Research Institute, College of Life Sciences Nanjing Forestry University Nanjing China; ^2^ Department of Mathematical Sciences, Centre for Invasion Biology Stellenbosch University Stellenbosch South Africa; ^3^ Mathematical and Physical Biosciences African Institute for Mathematical Sciences Cape Town South Africa; ^4^ State Key Laboratory of Desert and Oasis Ecology Key Laboratory of Ecological Security and Sustainable Development in Arid Lands, Xinjiang Institute of Ecology and Geography Urumqi, Xinjiang China

**Keywords:** adjusted Gini coefficient, diameter at breast height, skewness, Weibull distribution

## Abstract

Skewness, a measure of the asymmetry of a distribution, is frequently employed to reflect a biologically important property. Another statistic, the Gini coefficient (GC), originally used to measure economic inequality, has been validated in measuring the inequality of biological size distributions. Given that the GC and skewness control overlapping domains and interact with each other, researchers are perplexed by their relationship (varying with the biological [organ, tissue or cell] size distributions) and use both of them together to provide a more complete picture of the data. This study provides analytical forms of the GC for biological size distributions, including two‐parameter Weibull, uniform, normal, two‐parameter lognormal, gamma, three‐parameter Weibull, three‐parameter lognormal, and three‐parameter gamma distributions. Two empirical data sets and simulation data sets were used to clarify the GC–skewness relationships under different distributions. For the aforementioned distributions, the GC–skewness relationships can be divided into three types: (i) for a symmetrical distribution, the skewness is 0, and the GC ranges from 0.56 to 0.58 multiplied by the standard deviation divided by the mean irrespective of its relationship with the skewness; (ii) for an asymmetric distribution with a zero threshold, the GC is a monotonously increasing function of the skewness, and the two measures are equivalent; (iii) for an asymmetric distribution with a non‐zero threshold, the GC is determined by the skewness and an additional correction factor. We showed the differences in improving the accuracy of GC calculations based on small‐sample adjustments among various calculation methods, including the polygon (trapezoidal set) area method and the rotated Lorenz curve method. The present study turns the GC into a property of the distribution and offers a clear understanding for the GC–skewness relationship. This work provides insights into selecting and using the GC to measure inequality in ecological data, facilitating more accurate and meaningful analyses.

## Graphical abstract

This study provides an analytical solution of the Gini coefficient for Weibull distributions and uses real tree diameter at breast height data to clarify the relationship between the Gini coefficient and skewness. We propose a method for calculating the Gini coefficient and adjusting it for sample size under different distributions.

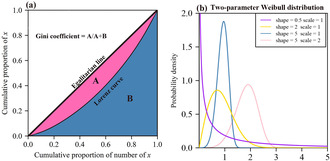

## 1 Introduction

The distribution of individual size and biomass reflects the physiological, ecological, and evolutionary characteristics of organisms. Changes in individual size and biomass frequency distributions with density, time, or external environmental factors can indicate the species' survival ability and growth strategies (Solbrig 1981; Huang, Ma, and Gladish 2024). For instance, the tradeoff between the mean size and density in a single population can reflect the effects of space and resource competition on plant growth and mortality (which can be reflected by the self‐thinning law) (Dillon et al. 2019). Individual size distributions can reflect the functional composition of a system and, at the whole community scale, are a key link between total abundance measures and ecological function (Diaz and Ernest 2024). Consequently, precise quantification of the frequency distribution of size or biomass is important, and various statistics have been established to measure or describe size or biomass frequency distribution characteristics, including the mean (center of the distribution), variance (dispersion around the mean), skewness (degree of asymmetry), kurtosis (pointiness), and Gini coefficient (inequality) (Hara 1988; Weiner and Thomas 1986).

Among these statistics, the Gini coefficient is often correlated with others, especially skewness. A large Gini coefficient often results from a large variance or skewness, and the Gini coefficient is sensitive to small changes in the mean of the distribution that do not concentrate around zero (Bendel et al. 1989; Weiner and Solbrig 1984). The relationship between inequality measures like the Gini coefficient, coefficient of variation, Theil index, and mean log deviation index has been discussed in detail using the empirical leaf area data of 240 individual plants of *Shibataea chinensis* Nakai, which shows that there are significant positive correlations between the Gini coefficient, coefficient of variation (CV), the square root of the Theil index, and the square root of the mean log deviation index (Huang et al. 2023). The half of CV^2^, Theil index, and mean log deviation index can be three special cases from a generalized entropy model in information theory for calculating the size inequality. However, the relationship between the Gini coefficient and skewness remains ambiguous due to varying relationships under different distributions the studied data conforming to.

Literature holds divided views on the Gini coefficient and skewness relationship. Some argue that inequality and skewness are distinct concepts and that inequality statistics are more relevant to variance than skewness, as non‐skewed distributions can also generate inequality (Kokko et al. 1999). Some focus on practicality, recommending different coefficients for various situations (Bendel et al. 1989). For example, the Gini coefficient may be highly positively correlated with skewness when the biological size data follow a two‐parameter lognormal distribution; however, skewness is recommended when the biological size data follow a three‐parameter lognormal distribution. Other researchers favor the Gini coefficient for its robustness and comprehensive reflection of size inequality, whereas skewness only describes the distribution shape (Weiner and Solbrig 1984; Weiner and Thomas 1986). For instance, Weiner and Solbrig (1984) proposed a definition of “size hierarchy” as follows: (1) individual sizes within a population vary widely; (2) there are fewer large individuals and more small individuals; (3) fewer large individuals contribute a disproportionately large amount of the population's biomass. They argued that skewness could only reflect the second of these categories and is inferior to the Gini coefficient in capturing size inequality (or size hierarchy) among members of plant populations. Our aim is to elucidate the relationship between the Gini coefficient and skewness and promote their use in ecology.

This study examined the relationship between the Gini coefficient and skewness under various common statistical distributions of biological (organ, tissue, and cell) size including Weibull, uniform, normal, lognormal, gamma distributions, with a detailed analysis of the two‐parameter Weibull distribution, a continuous probability distribution accommodating various shapes, often used in reliability analysis and modeling skewed size distributions in ecology (Weibull 1951; Taubert et al. 2013). We provided expressions of the Gini coefficient under Weibull, uniform, normal, lognormal, gamma distributions and a method for calculating the Gini coefficient when the data follow one of these distributions. The method is validated using tree diameter at breast height data, commonly modeled by the two‐parameter Weibull distribution, and the simulation data generated from the various distributions mentioned above (Teimouri, Abdolahnezhad, and Ghalandarayeshi 2020). We proposed a correction factor to the Gini coefficient for the distributions with a non‐zero threshold parameter, tested the validity of this method using the seedhead length data of *Setaria viridis* (L.) P. Beauv. to examine whether the empirical data follow a three‐parameter lognormal distribution.

## 2 Definitions and Properties of the Statistics

### 2.1 Lorenz Curve

The Lorenz curve is widely used as a powerful device for examining the inequality of the size distribution, and it is generally defined as (Lorenz 1905):

(1) $$\eta =L\left(\pi \right),0\leq \pi \leq 1$$

where *π* is the cumulative proportion of individuals ranked according to size, and *η* is the cumulative proportion of the corresponding individual size. Suppose individual size *x* follows a certain cumulative probability distribution *F*(*x*) with support in the positive real numbers and a finite expectation *μ*. In that case, the Lorenz curve can be further defined as (Sarabia 1997):

(2) $$L_{F}\left(\pi \right)=\mu^{-1}\int_{0}^{\pi }F^{-1}\left(p\right)\mathrm{dp},0\leq \pi \leq 1$$

where *F* ^−1^(*p*) is the quantile function for *F*(*x*), and *p* is the quantile (0≤p≤1). The Lorenz curve is a concave upward curve that passes through the points (0, 0) and (1, 1). The closer the curve is to the egalitarian line connecting these points, the more equitable the size distribution.

### 2.2 Gini Coefficient

**FIGURE 1:**
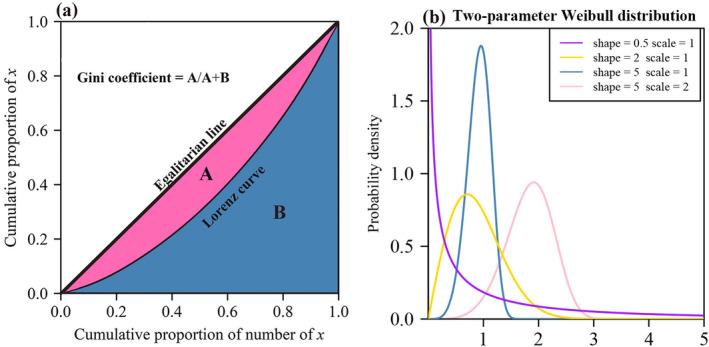
The Lorenz curve and the Gini coefficient for a hypothetical sample {*x*_*i*_} (a) and the illustrative curves of the Weibull distribution for different shape and scale parameters (b).

When two Lorenz curves intersect, comparing relative size inequality visually is difficult. As such, the Gini coefficient quantifies size inequality by calculating the ratio of the area between the Lorenz curve and the egalitarian line to the area below the egalitarian line (Gini 1912) (see Figure 1a):

(3) $$G=1-2\int_{0}^{1}L\left(\pi \right)\mathrm{d\pi}$$

Given the Lorenz curve under cumulative size distribution *F*(*x*) and the definition of the Gini coefficient, we can express the Gini coefficient for a specific cumulative size distribution as:

(4) $$G_{F}=1-2\mu^{-1}\int_{0}^{1}\int_{0}^{\pi }F^{-1}\left(p\right)\mathrm{dpd\pi}$$

### 2.3 Two‐Parameter Weibull Distribution

For *x* > 0, the cumulative distribution *F*(*x*) and probability density functions *f*(*x*) of the two‐parameter Weibull distribution are (Weibull 1951):

(5) $$F\left(x\right)=1-e^{-{\left(\frac{x}{\alpha }\right)}^{\beta }}$$

and

(6) $$f\left(x\right)=\frac{\beta }{\alpha }{\left(\frac{x}{\alpha }\right)}^{\beta -1}e^{-{\left(\frac{x}{\alpha }\right)}^{\beta }}$$

where *β* > 0 (shape parameter) controls the distribution shape, and *α* > 0 (scale parameter) controls the distribution size. When *β* < 1, the Weibull distribution is extremely right‐skewed. When 1 < *β* < 3.6, the Weibull distribution is right‐skewed. When *β* > 3.6, the Weibull distribution is left‐skewed. When *β* ≈ 3.6, the Weibull distribution approximates normality. The quantile function for the two‐parameter Weibull distribution is (Lai, Murthy, and Xie 2011; Lai 2014) (see Figure 1b):

(7) $$F^{-1}\left(p\right)=\alpha {\left[-\ln\left(1-p\right)\right]}^{\frac{1}{\beta }}$$

The mean of the two‐parameter Weibull distribution is:

(8) $$E\left(x\right)=\alpha \Gamma \left(1+\frac{1}{\beta }\right)$$

and

(9) $$\Gamma \left(k\right)=\int_{0}^{\infty }t^{k-1}e^{-t}\mathrm{dt}$$

### 2.4 Moment Skewness

Skewness *S* measures the degree of asymmetry of a cumulative distribution *F*(*x*) by calculating the third‐order moments of the standard score of *x*:

(10) $$S=E\left[{\left(\frac{x-\mu }{\sigma }\right)}^{3}\right]=\frac{\mu_{3}}{{\mu_{2}}^{3/2}}$$

where *μ*_2_ and *μ*_3_ are the second and third central moments of the distribution, for the two‐parameter Weibull distribution, skewness *S* is given by (Lai, Murthy, and Xie 2011; Lai 2014):

(11) $$S_{W}=\frac{\Gamma \left(1+\frac{3}{\beta }\right)-3\Gamma \left(1+\frac{1}{\beta }\right)\Gamma \left(1+\frac{2}{\beta }\right)+2\Gamma^{3}\left(1+\frac{1}{\beta }\right)}{{\left[\Gamma \left(1+\frac{2}{\beta }\right)-\Gamma^{2}\left(1+\frac{1}{\beta }\right)\right]}^{\frac{3}{2}}}$$

## 3 Materials and Methods

### 3.1 Calculating the Sample Skewness and Sample Gini Coefficient

Various measures of sample skewness (*s*) are defined and adopted by many software packages. Fisher‐Pearson's coefficient of skewness is the most traditional one that replaces the population central moment with the sample central moment. However, the sample central moment is not an unbiased estimate of the population central moment. The adjusted Fisher‐Pearson coefficient of skewness, which replaces the population central moment with the adjusted unbiased sample central moment, addresses this issue (Joanes and Gill 1998). Suppose *x* = (*x*_1_, *x*_2_, …, *x*_*n*_) is a sample of size *n*. The adjusted Fisher‐Pearson coefficient of skewness is defined as:

(12) $$s=\frac{\sqrt{n\left(n-1\right)}}{n-2}\frac{m_{3}}{{m_{2}}^{3/2}}$$

where *m*_*r*_ is the *r*‐th central moment of the sample and is given by:

(13) $$m_{r}=\frac{1}{n}\sum_{i}^{n}{\left(x_{i}-\bar{x}\right)}^{r}$$

*s* is an unbiased estimate of the population skewness for normal samples, but for samples from non‐normal distributions, *s* is biased. Various measures of sample skewness have been proposed, each with its own advantages and disadvantages. No single measure has the smallest root mean square error across all distributions (Joanes and Gill 1998). The adjusted Fisher‐Pearson coefficient of skewness is one of the more widely used measures adopted by software packages such as SAS and R.

Calculating the sample Gini coefficient (*g*) involves determining the area between the Lorenz curve and the egalitarian line. For a sample *x* = (*x*_1_, *x*_2_, …, *x*_*n*_), this area forms a polygon. The Gini coefficient is equal to twice the area of this polygon. We used the “area” function in the spatstat.geom package (Baddeley, Rubak, and Turner 2015) in R software (version 4.3.3; R Core Team 2024) to calculate the polygon's area. The area of the polygon is also equivalent to 1/2 minus the area below the Lorenz curve, which is the sum of *n* trapezoids. The *i‐*th trapezoid has height 1/*n* and bases *η*_*i*–1_ and *η*_*i*_, where *η*_0_ = 0, *η*_*n*_ = 1, and *η*_*i*_ is the cumulative proportion of all individuals whose size is less than or equal to that of the *i‐*th individual. However, the sample Gini coefficient is affected by the sample size. For example, the Gini coefficient of a small sample would be smaller than that of a large sample generated by the same stochastic process. When the sample size is sufficiently large, the sample Gini coefficient can approximate the population Gini coefficient. An adjustment based on sample size can reduce this small‐sample bias (Deltas 2003; Huang et al. 2023):

(14) $$g=1-\frac{1}{n}\sum_{i=1}^{n}\left(\eta_{i-1}+\eta_{i}\right)=\frac{\sum_{i=1}^{n}\sum_{j=1}^{n}\left|x_{j}-x_{i}\right|}{2n^{2}\bar{x}}$$

and

(15) $$g_{\mathrm{adj}}=\frac{n}{n-1}g$$

where *g* is the sample Gini coefficient calculated from the area of the polygon enclosed by the Lorenz curve and the egalitarian line, and *g*_adj_ is the adjusted sample Gini coefficient.

### 3.2 Data of Size Distributions

Two empirical data sets of organ size distributions to test the validity of the theoretical expressions for the Gini coefficient in practical applications and its relationship with skewness.

- Data set 1: We used a forest census data set previously collected within a 400 m × 1000 m study region in the Beijing Songshan National Nature Reserve, China (40°30′50″ N, 115°49′12″ E), surveyed in August 2014 (see Shi et al. [2018] for details); data were accessible in Shi et al. (2023). The Latin names of species and the diameter at breast height (DBH) values of all trees with a DBH ≥ 1 cm in the study region were recorded. The study region was divided into 160 quadrats of 50 m × 50 m, resulting in 160 groups of DBH data (see Table S1).
- Data set 2: We collected the seedhead length data of *S. viridis* (L.) P. Beauv. in the 15 m × 15 m study area in September 2015. The study area is located in a field of Whitehouse Experiment Station of Nanjing Forestry University (31°37′55′′ N, 119°07′42′′ E). The study area was divided into 125 quadrats of 1 m × 1 m, resulting in 125 groups of *S. viridis* seedhead length data (see Table S3).

### 3.3 Parameter Estimation and Statistical Test of Size Distributions

Maximum likelihood estimation (MLE) is a statistical method for estimating the parameters of a probability distribution that best describes a given dataset. Assuming that the DBH data is drawn from a two‐parameter Weibull distribution, the shape and scale parameters of the Weibull distribution can be estimated using MLE. The *S. viridis* seedhead length data were handled in a similar way, assuming drawn from a three‐parameter lognormal distribution, and then the shape, scale, and threshold parameters of the lognormal distribution can be estimated using MLE. We used the “mle2” function in the “bbmle” package (Bolker 2023) in R software (version 4.3.3; R Core Team 2024) to estimate the parameters of the size distribution.

The Kolmogorov–Smirnov test (Lopes 2011) is a non‐parametric test used to determine whether two samples are from the same population or to decide if a sample comes from a population with a specific distribution. This test assesses whether there is a significant difference between the compared samples by calculating the maximum difference between the cumulative distribution functions (CDFs) of the two samples. This study used the Kolmogorov–Smirnov test to determine whether the DBH data ( *S. viridis* seedhead length data) came from a two‐parameter Weibull distribution (three‐parameter lognormal distribution), with its parameters estimated by MLE (see the previous paragraph for details).

The Wilcoxon signed‐rank test (Wilcoxon 1945) is a nonparametric statistical hypothesis test used to assess the position of a group of samples or compare the relative positions of two matched samples. Specifically, it evaluates the median difference between two paired samples. When the data are skewed and do not meet the assumption of normality, the Student's *t*‐test (Student 1908) is not appropriate. For instance, if the data fail to pass the Shapiro–Wilk test (Shapiro and Wilk 1965), which tests for normality, the *t*‐test should not be used. In such cases, the Wilcoxon signed rank test is a suitable alternative. In this study, we used the Wilcoxon signed‐rank test to test the significance of the difference between the adjusted sample Gini coefficients and the population Gini coefficients. The Wilcoxon signed‐rank test was used because the tested data failed to pass the Shapiro–Wilk test.

Multivariate analysis of variance (MANOVA) is a generalization of the *t*‐test and analysis of variance (ANOVA). It extends the capabilities of ANOVA by simultaneously analyzing the differences between group means for multiple dependent variables. In this study, we used MANOVA to test the significance of the differences between group means of sample Gini coefficients and sample skewness under different scale parameters.

### 3.4 Gini Coefficient–Skewness Relationship of Two‐Parameter Weibull Distributions

Based on the properties of two‐parameter Weibull distributions and the expression for the Gini coefficient under this specific distribution, we derived a specific formula for the Gini coefficient of the two‐parameter Weibull distributions:

(16) $$G_{W}=1-2\mu^{-1}\int_{0}^{1}\int_{0}^{\pi }F^{-1}\left(p\right)\mathrm{dpd\pi}=1-{\left(\frac{1}{2}\right)}^{\frac{1}{\beta }}$$

Evidently, the Gini coefficient of the two‐parameter Weibull distribution decreases with the shape parameter but is not influenced by the size parameter. Similarly, the skewness of the two‐parameter Weibull distribution is unaffected by the size parameter and is solely determined by the shape parameter. By analyzing the expressions for the Gini coefficient and skewness of the two‐parameter Weibull distribution, we derived their functional relationship in the following form:

(17) $$S_{W}=\frac{\Gamma \left(1+3\log_{0.5}\left(1-G_{W}\right)\right)-3\Gamma \left(1+\log_{0.5}\left(1-G_{W}\right)\right)\Gamma \left(1+2\log_{0.5}\left(1-G_{W}\right)\right)+2\Gamma^{3}\left(1+\log_{0.5}\left(1-G_{W}\right)\right)}{{\left[\Gamma \left(1+2\log_{0.5}\left(1-G_{W}\right)\right)-\Gamma^{2}\left(1+\log_{0.5}\left(1-G_{W}\right)\right)\right]}^{\frac{3}{2}}}$$

The numerical derivative of Equation (17) on the interval (0, 1) is greater than 0. This derivative was calculated using the accurate numerical derivative calculation method implemented in the “grad” function of the “numDeriv” package (Gilbert and Varadhan 2019) in R software (version 4.3.3; R Core Team 2024). We computed the numerical roots of Equation (17) as 0.1750364 using the “uniroot” function of the “stats” package (Brent 1973) in R (version 4.3.3; R Core Team 2024), which means that it is feasible to determine whether the data are right‐skewed by calculating whether the Gini coefficient is greater than 0.1750364 in the event that the data conforms to a two‐parameter Weibull distribution.

### 3.5 Data Validation Process

We conducted 1000 random samplings with 1000 random numbers for each sampling from the two‐parameter Weibull distribution, characterized by the shape parameter *β* and the scale parameter *α*. Each sample's *β* was randomly drawn from a uniform distribution on the interval (0, 10), while *α* was drawn from a uniform distribution on the interval (0, 1000). The *β* interval was set to ensure that our samples covered cases of left‐skewed, right‐skewed, and approximately normal distributions. For each sample, the shape parameter was recorded to compute population skewness (Equation 11) and the population Gini coefficient (Equation 16), while the generated sample was used to compute sample skewness (Equation 12) and the adjusted sample Gini coefficient (Equation 15).

We used the Kolmogorov–Smirnov test to determine whether the DBH data followed a Weibull distribution. Out of 160 DBH data sets, 136 passed the test. The shape and scale parameters of the two‐parameter Weibull distribution for these 136 data sets were estimated by maximizing the likelihood function (Table S2). The Gini coefficient can be estimated as:

(18) $$\hat{g}W=1-{\left(\frac{1}{2}\right)}^{\frac{1}{\hat{\beta }}}$$

We calculated the estimated Gini coefficients and adjusted sample Gini coefficients (Equations 18 and 15) for the 136 DBH data sets (Table S2).

## 4 Results Under Two‐Parameter Weibull Distribution

**FIGURE 2:**
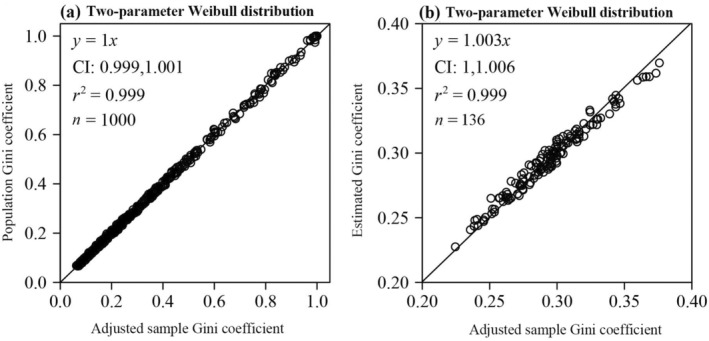
Linear fits to the population Gini coefficient vs. the adjusted sample Gini coefficient for 1000 simulated samples from the two‐parameter Weibull distribution (a), and linear fits to the estimated Gini coefficient vs. the adjusted sample Gini coefficient for 136 DBH data sets (b). Here, *y* denotes the population (or estimated) Gini coefficient calculated by Equation (16) (or Equation 18), and *x* denotes the adjusted sample Gini coefficient calculated by Equation (15); CI represents the 95% confidence interval of the slope; *r* ^2^ represents the coefficient of determination.

There was no statistically significant difference between the adjusted sample Gini coefficient (*g*_adj_) and the population Gini coefficient (*G*_*W*_), as the 95% confidence interval (CI) for *G*_*W*_ vs. *g*_adj_ included unity (Figure 2a), and the *p*‐value of the paired Wilcoxon signed rank test was greater than 0.05.

**FIGURE 3:**
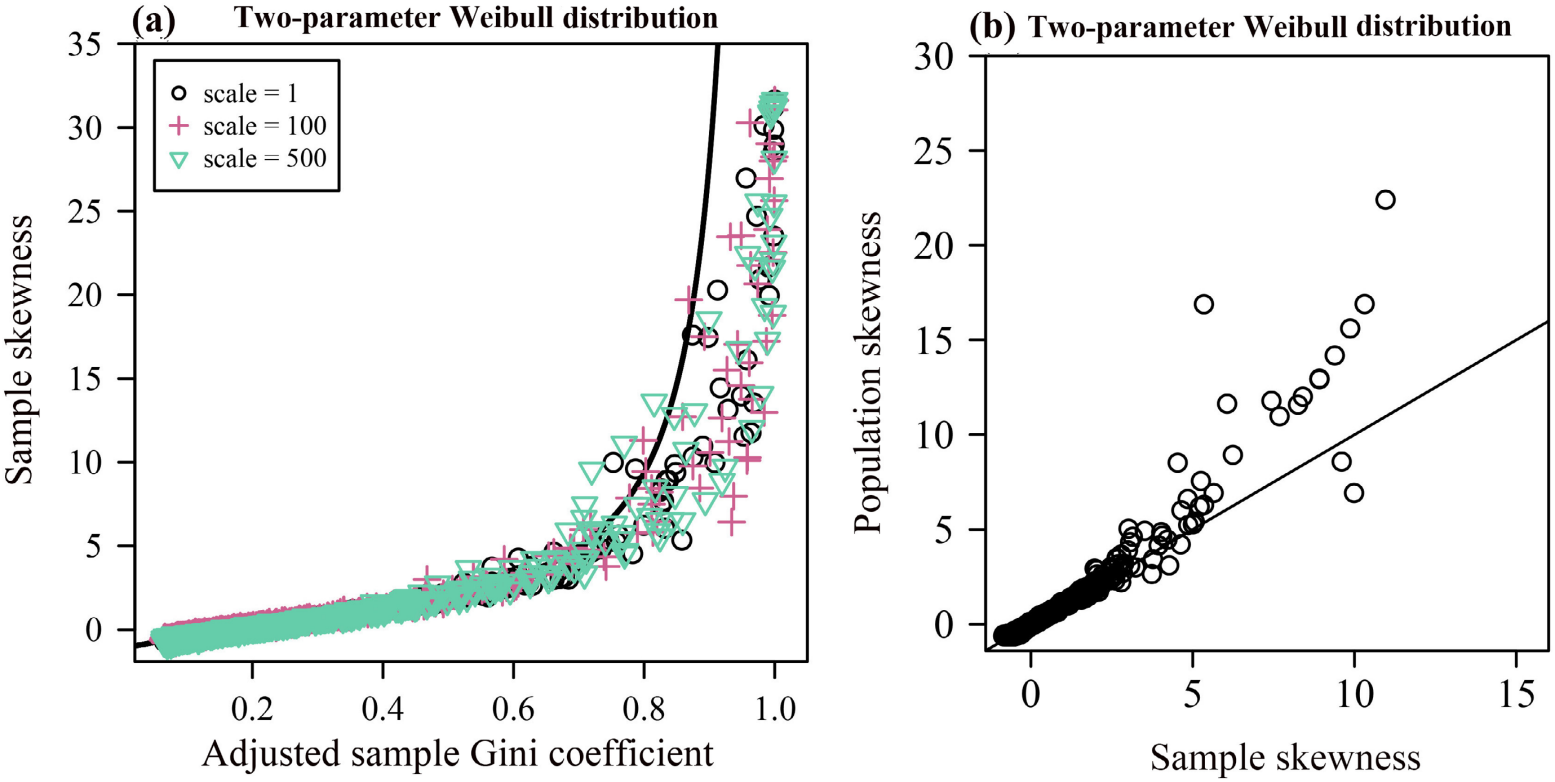
Scatterplot of the adjusted sample Gini coefficient and sample skewness calculated from 1000 simulated samples from the Weibull distribution for three different scale parameters (a), and scatterplot of the sample skewness and population skewness calculated from 1000 simulated samples from the two‐parameter Weibull distribution for a fixed size parameter (*α* = 1) (b). In each panel, the symbols represent the simulated samples, the different shapes and colors of the symbols represent the simulated samples drawn from a Weibull distribution with different scale parameters, the curve represents the function curve between the Gini coefficient and skewness under the Weibull distribution (Equation 17), the line is a straight line that crosses the origin with a slope of 1, *x* represents the sample Gini coefficient calculated by the polygonal (trapezoidal sets) area (a) (or sample skewness calculated by Equation 12 (b)), and *y* represents the sample skewness calculated by Equation (12) (a) (or population skewness calculated by Equation 11 (b)).

**FIGURE 4:**
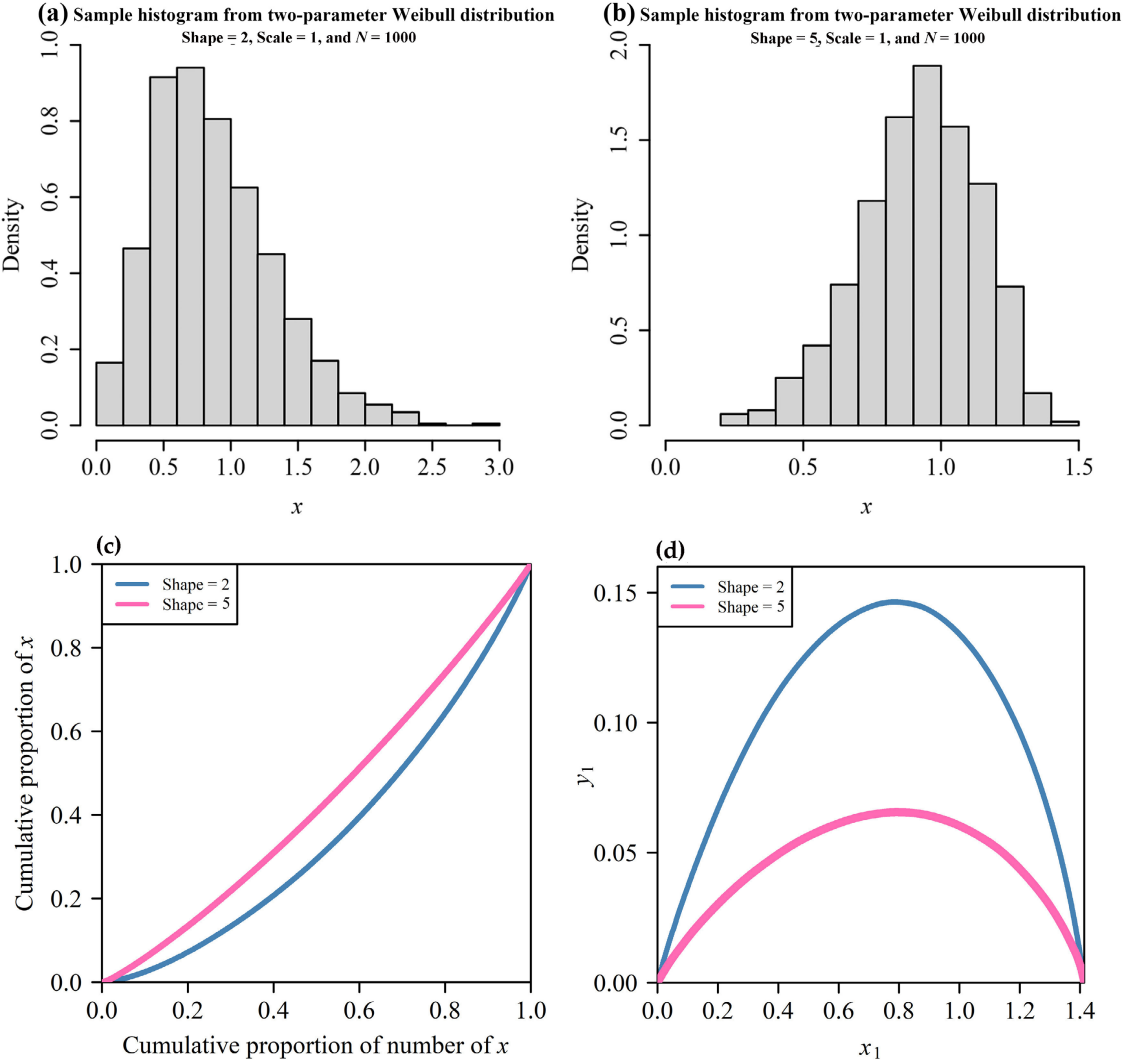
Histograms of two groups of samples from Weibull distributions with representative shape parameters. (a) Shape parameter = 2, indicating positive skewness, and (b) shape parameter = 5, indicating negative skewness. The Lorenz curves of those samples are depicted in (c), and their rotated versions are shown in (d), illustrating the effect of skewness on the Lorentz curve under the Weibull distribution. In panel (d), the rotated curves shown in red and blue fit with the performance equations. The Gini coefficient is equal to twice the area under the rotated curve.

Figure 3a shows the relationship between the Gini coefficient and skewness, demonstrating that the Gini coefficient increases with skewness under two‐parameter Weibull distribution. When the distribution is right‐skewed (positive skewness), it indicates more poor households and a few extremely rich households (resulting in a large Gini coefficient). Conversely, when the distribution is left‐skewed (negative skewness), it indicates more rich households and a few poor households (resulting in a small Gini coefficient) (see Figure 4). Figure 3a also shows that the scale parameter does not affect the relationship between sample skewness and the sample Gini coefficient (the *p*‐value of the MANOVA was greater than 0.05). The deviation of data points from the curve (Equation 17) at larger values of population skewness occurs because the sample skewness estimate is biased when the sample deviates from normality (see Figure 3b). Figure 3b shows that sample skewness is smaller than population skewness when population skewness exceeds 5.

There was a negligible difference between the estimated Gini coefficients (${\hat{g}}_{W}$) and the adjusted sample Gini coefficient, as the 95% confidence interval (CI) of ${\hat{g}}_{W}$ vs. *g*_adj_ included unity (Figure 2b), providing a new method to calculate the Gini coefficient for the data comforting to the two‐parameter Weibull distribution.

## 5 The Gini Coefficients for Other Distributions

The relationship between the Gini coefficient and skewness varies across size distributions. For example, in an exponential distribution, the Gini coefficient is fixed at 0.5, and the skewness is fixed at 2 (Bendel et al. 1989). We thus discuss the relationship between the Gini coefficient and skewness under other size distributions.

### 5.1 Uniform Distribution

The Gini coefficient for a uniform distribution defined on the interval $\left[a,b\right]\;\left(a>0\right)$ is:

(19) $$G_{U}=1-2\mu^{-1}\int_{0}^{1}\int_{0}^{\pi }F^{-1}\left(p\right)\mathrm{dpd\pi}=\frac{b-a}{3\left(b+a\right)}=\frac{\sigma }{\sqrt{3}\mu }\approx \frac{\;0.5773503\sigma }{\mu }$$

where *μ* is the expectation of the uniform distribution, *σ* is the standard deviation of the uniform distribution. Since *a*, *b* > 0, $G_{U}=\frac{b–a}{3\left(b+a\right)}=\frac{1}{3}\left(1–\frac{2a}{b+a}\right)<\frac{1}{3}$.

**FIGURE 5:**
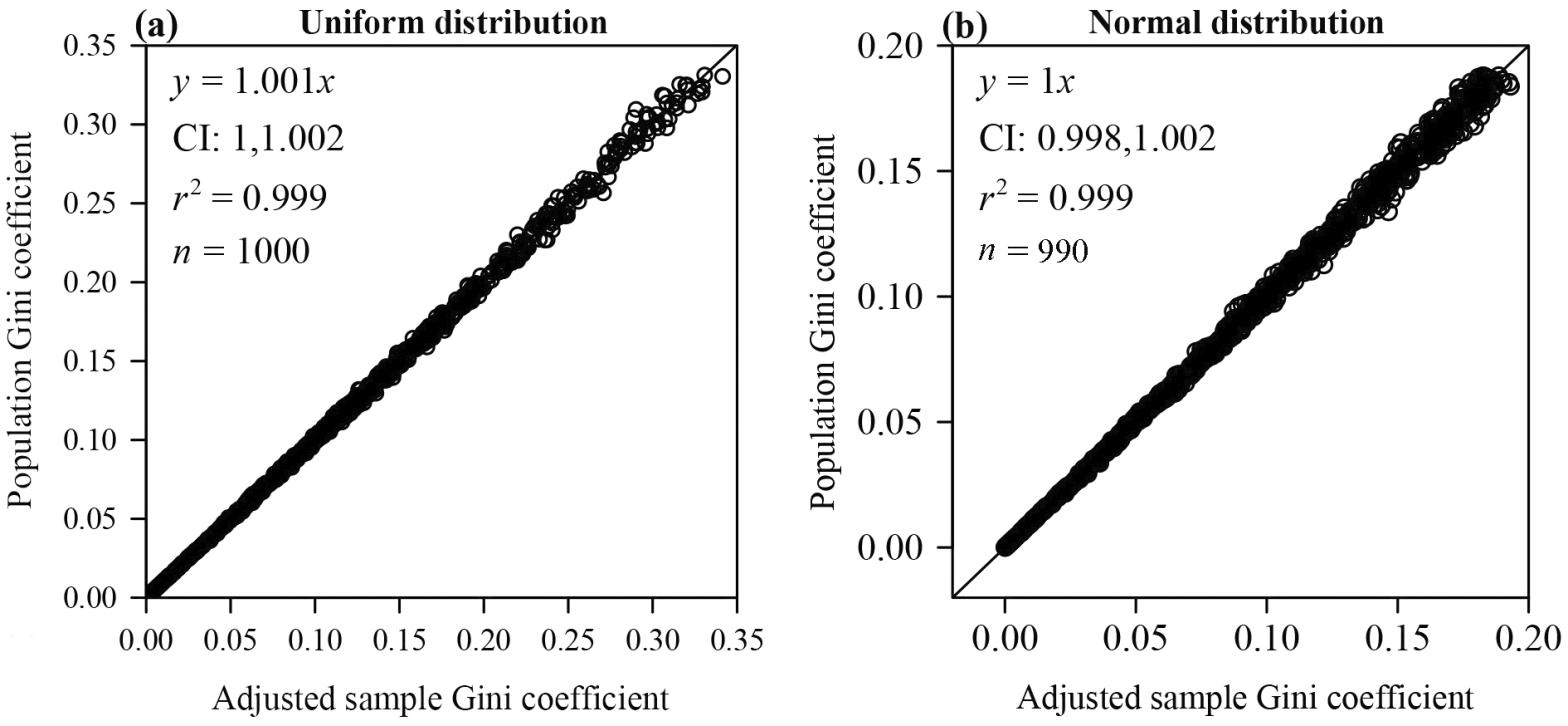
Linear fits to the population Gini coefficient vs. the adjusted sample Gini coefficient for simulated data drawn from the uniform (a) and normal distributions (b). Here, *y* denotes the population Gini coefficient calculated by Equations (19) and (20), and *x* denotes the adjusted sample Gini coefficient calculated by Equation (15); CI represents the 95% confidence interval of the slope; *r* ^2^ represents the coefficient of determination; the straight line represents the regression line.

We conducted 1000 random samplings with 1000 random numbers for each sampling from the uniform distribution defined by *a* and *b*. The parameter *a* of each sample was drawn from a uniform distribution on (0, 500), and the parameter *b* was drawn from a uniform distribution on (0, 500). There was no statistically significant difference between the adjusted sample Gini coefficient (*g*_adj_, calculated by Equation 15) and *G*_*U*_ (calculated by Equation 19), as the 95% CI of *G*_*U*_ versus *g*_adj_ included unity, and the *p*‐value of the paired Wilcoxon signed‐rank test was greater than 0.05 (Figure 5a).

### 5.2 Normal Distribution

Since biological size measures cannot be negative, the size distribution *F*(*x*) should be supported within the subset of positive real numbers. If a normal distribution is used as the size distribution, at least *μ* − 3*σ* > 0 must be ensured because there is a 99.86% chance that *x* is greater than 0. The Gini coefficient of a normal distribution can be estimated as

(20) $$G_{N}=1-2\mu^{-1}\int_{0}^{1}\int_{0}^{\pi }F^{-1}\left(p\right)\mathrm{dpd\pi}=\frac{2\sigma }{\mu }\int_{0}^{1}\Phi^{-1}\left(p\right)\mathrm{pdp}\approx \frac{0.5641896\sigma }{\mu }$$

where *μ* is the expectation of the normal distribution, *σ* is the standard deviation of the normal distribution, Ф(*x*) denotes the standard normal distribution function, and Ф^−1^(*p*) is the standard normal quantile function. Since Ф^−1^(*p*) can be calculated using standard mathematical and statistical software packages (e.g., R software), the term $\int_{0}^{1}1–{Ф}^{–1}\left(p\right)\mathrm{pdp}$ can be calculated to be approximately equal to 0.2820948. Therefore, *G*_*N*_ is approximated as 0.5641896*σ*/*μ*. Given that *μ* − 3*σ* > 0, i.e., *σ*/*μ* < 1/3, *G*_*N*_ is less than 0.1880632.

We conducted 1000 random samplings with 1000 random numbers for each sampling from the normal distribution defined by the mean *μ* and the standard deviation *σ*. The mean *μ* and standard deviation *σ* of each sample were drawn from a bivariate uniform distribution on a domain of support *D* satisfying $x\;\epsilon \;\left(0,300\right),y\;\epsilon \;\left(0,100\right),\text{and}\;x>3y$. There was no statistically significant difference between the adjusted sample Gini coefficient (*g*_adj_, calculated by Equation 15) and *G*_*N*_ (calculated by Equation 20), as the 95% CI of *G*_*N*_ versus *g*_adj_ included unity, and the *p*‐value of the paired Wilcoxon signed‐rank test was greater than 0.05 (Figure 5b). The skewness of the normal distribution is fixed at 0. It is not affected by the mean or standard deviation, indicating that the relationship between the Gini coefficient and skewness indeed varies across different distributions.

### 5.3 Two‐Parameter Lognormal Distribution

The lognormal distribution with a positive range, right skewness, heavy right tail, and easy‐to‐calculate parameter estimates is frequently used as a population growth model, as well as for species abundance modeling (Crow 1988). The probability density function (PDF), *f*(*x*), of a two‐parameter lognormal distribution is given by Mateu‐Figueras and Olea (2022):

(21) $$f\left(x\right)=\frac{1}{\sqrt{2\pi }\mathrm{\sigma x}}\exp\left\{-\frac{{\left[\ln\left(x\right)-\mu \right]}^{2}}{2\sigma^{2}}\right\},x\in \left(0,\infty \right)$$

where *μ* and *σ* are the mean and standard deviation of the logarithm of the variable. The quantile function for the two‐parameter lognormal distribution is given by Mateu‐Figueras and Olea (2022):

(22) $$F^{-1}\left(p\right)=\exp\left[\mu +\sigma \Phi^{-1}\left(p\right)\right],0\leq p\leq 1$$

The mean of the two‐parameter lognormal distribution is given by:

(23) $$E\left(x\right)=\exp\left(\mu +\frac{1}{2}\sigma^{2}\right)$$

Based on the properties of the lognormal distribution mentioned above, the Gini coefficient of the two‐parameter lognormal distribution can be expressed as:

(24) $$\begin{array}{c} G_{L}=1-2\mu^{-1}\int_{0}^{1}\int_{0}^{\pi }F^{-1}\left(p\right)\mathrm{dpd\pi}\\ =1-2\int_{-\infty }^{\infty }\Phi \left(x-\sigma \right)\varphi \left(x\right)\mathrm{dx}=1-2E\left(\Phi \left(x-\sigma \right)\right) \end{array}$$

where Ф(*x*) denotes the standard normal distribution function; $\varphi \left(x\right)$ is the standard normal probability density function. The absence of *μ* in the expression for the Gini coefficient indicates that the Gini coefficient under the two‐parameter lognormal distribution is only affected by *σ* (the shape parameter).

**FIGURE 6:**
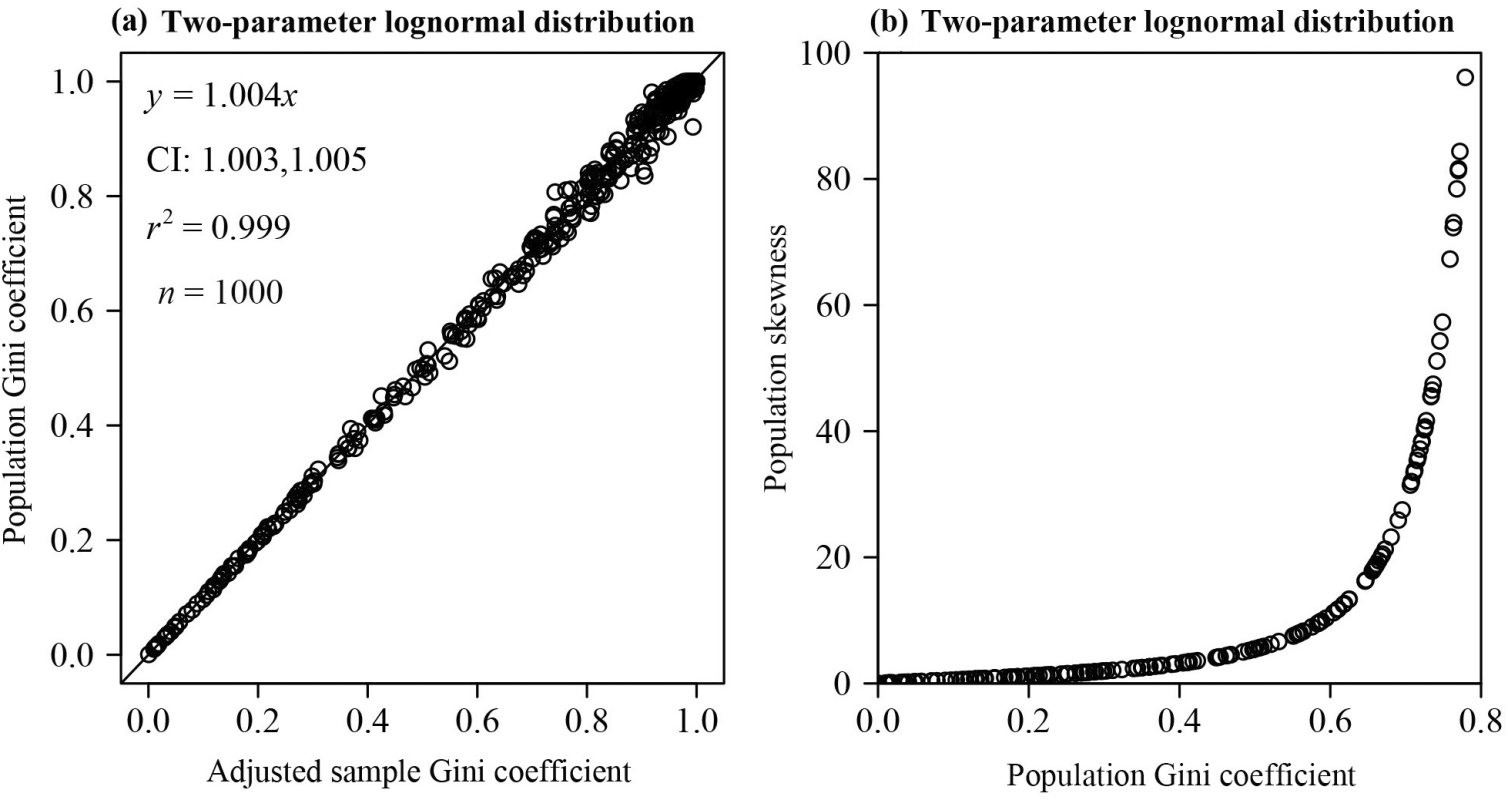
Linear fits to the population Gini coefficient vs. the adjusted sample Gini coefficient for simulation data drawn from two‐parameter lognormal distribution (a), and scatterplot of population Gini coefficient and population skewness calculated from simulated data drawn from two‐parameter lognormal distribution (b). In each panel, the open circles represent the simulated samples, *y* denotes the population Gini coefficient calculated by Equation (24) (a) (or the population skewness calculated by Equation 26 (b)), and *x* denotes the adjusted sample Gini coefficient calculated by Equation (15) (a) (or the population Gini coefficient calculated by Equation 24 (b)); *r* ^2^ represents the coefficient of determination, the straight line represents the regression line.

We conducted 1000 random samplings with 1000 random numbers for each sampling from the two‐parameter lognormal distribution defined by *μ* and *σ*. The *μ* of each sample was drawn from a uniform distribution on (0, 1000), and *σ* was drawn from a uniform distribution on (0, 10). Figure 6a illustrates a scatterplot of the adjusted sample Gini coefficient of the two‐parameter lognormal distribution (calculated by Equation 15) against the population Gini coefficient of the two‐parameter lognormal distribution (calculated by Equation 24).

The derivative of the Gini coefficient for the two‐parameter lognormal distribution can be computed as:

(25) $$\frac{{\mathrm{dG}}_{L}}{\mathrm{d\sigma}}=\int_{-\infty }^{\infty }\varphi \left(x-\sigma \right)\varphi \left(x\right)\mathrm{dx}=E\left[\varphi \left(x-\sigma \right)\right]>0$$

The skewness of the two‐parameter lognormal distribution is given by:

(26) $$S_{L}=\left(e^{\sigma^{2}}+2\right)\sqrt{e^{\sigma^{2}}-1}$$

The skewness of the two‐parameter lognormal distribution is a monotonic increasing function of the standard deviation parameter, and its Gini coefficient is also a monotonic increasing function of the standard deviation parameter. Consequently, the Gini coefficient of the two‐parameter lognormal distribution increases with increasing skewness (see Figure 6b). It is apparent that from Equation (26) the skewness is equal to 0 when the shape parameter *σ* is 0. Through Equation (24), the Gini coefficient was calculated to equal 0 at this point as well using the “integrate” function of the “stats” package (Piessens et al. 1983) in R (version 4.3.3; R Core Team 2024), which means that it is feasible to determine whether the data are right‐skewed by calculating whether the Gini coefficient is greater than 0 in the event that the data conforms to a two‐parameter lognormal distribution.

### 5.4 Gamma Distribution

The gamma distribution is a continuous probability distribution with two positive parameters *k* and *b*. It is commonly used to describe right‐skewed data. The probability density function (PDF), *f*(*x*), of the gamma distribution is given by (Jambunathan 1954):

(27) $$f\left(x\right)=\frac{1}{\Gamma \left(k\right)b^{k}}x^{k-1}e^{-\frac{x}{b}},x\in \left(0,\infty \right)$$

where *k* is the shape parameter of the gamma distribution, and *b* is the scale parameter of the distribution.

The mean of the gamma distribution is given by:

(28) $$E\left(x\right)=\frac{1}{\mathrm{bk}}$$

The Gini coefficient of the gamma distribution can be calculated as:

(29) $$G_{G}=1-2\mu^{-1}\int_{0}^{1}\int_{0}^{\pi }F^{-1}\left(p\right)\mathrm{dpd\pi}=1-2\frac{\int_{0}^{\infty }x^{k-1}e^{-x}\Gamma \left(k+1,x\right)\mathrm{dx}}{\Gamma \left(k\right)\Gamma \left(k+1\right)}$$

and

(30) $$\Gamma \left(k,x\right)=\int_{0}^{x}t^{k-1}e^{-t}\mathrm{dt}$$

The skewness of the gamma distribution is given by:

(31) $$S_{G}=\frac{2}{\sqrt{k}}$$

**FIGURE 7:**
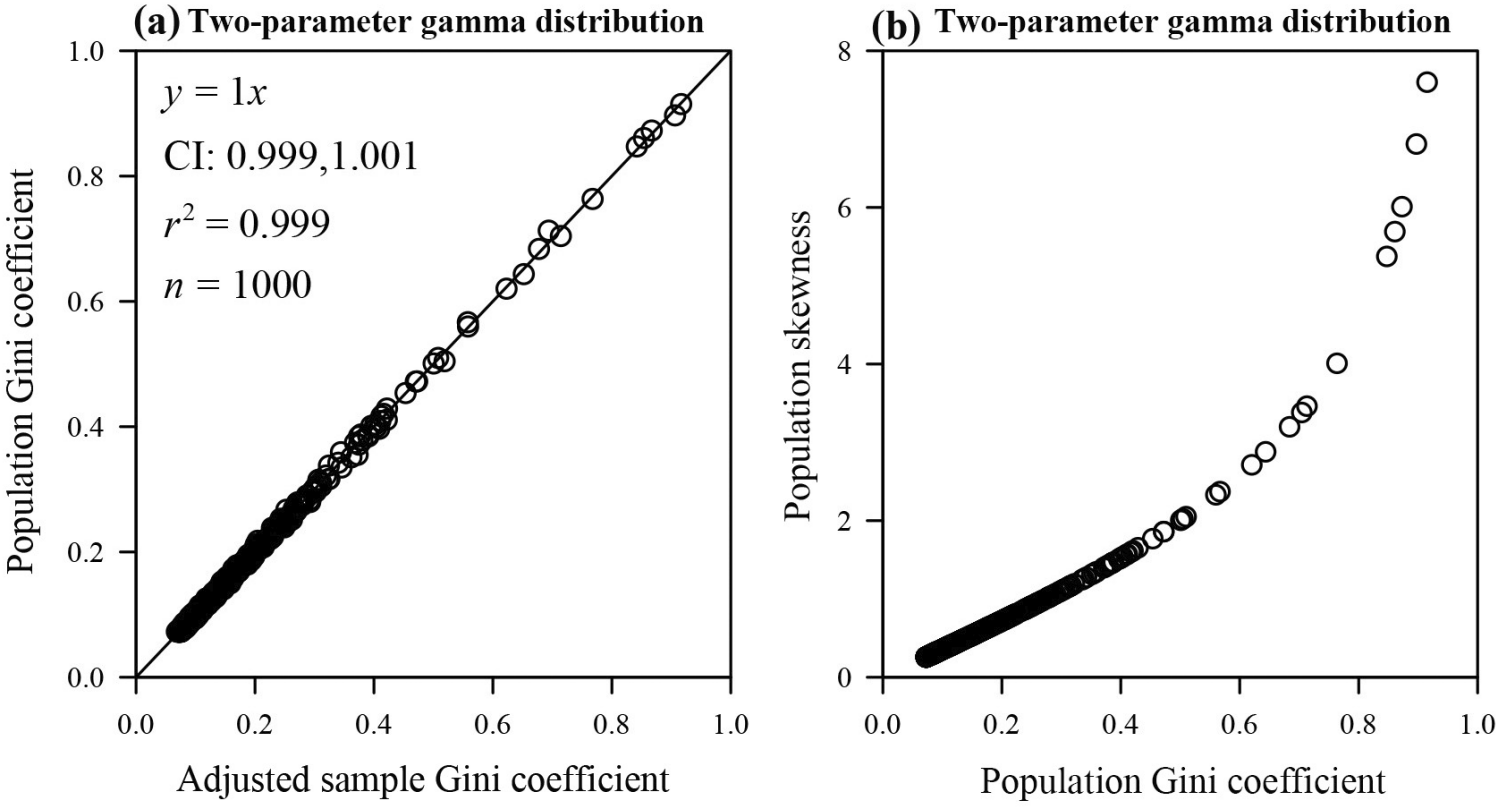
Linear fits to the population Gini coefficient vs. the adjusted sample Gini coefficient for simulated data drawn from two‐parameter gamma distribution (a), and scatterplot of population Gini coefficient and population skewness calculated from simulated data drawn from two‐parameter gamma distribution (b). In each panel, the open circles represent the simulated samples, *y* denotes the population Gini coefficient calculated by Equation (29) (a) (or the population skewness calculated by Equation 31 (b)), and *x* denotes the adjusted sample Gini coefficient calculated by Equation (15) (a) (or the population Gini coefficient calculated by Equation 29 (b)); *r* ^2^ represents the coefficient of determination, the straight line represents the regression line.

From the expression of *G*_*G*_, it is apparent that it is a function of the shape parameter *k* independent of the scale parameter *b*, and we calculated the numerical derivative of *G*_*G*_ and found that it was negative (given that *k* is generally less than 1000 in practical applications, we only calculated the case where *k* was less than 1000), which indicates that *G*_*G*_ is a monotonically decreasing function of *k*, and the skewness of the gamma distribution is also a monotonically decreasing function of *k*. Therefore, the Gini coefficient is a monotonically increasing function of skewness under the gamma distribution (see Figure 7b). We conducted 1000 random samplings with 1000 random numbers for each sampling from the gamma distribution defined by the parameters of *k* and *b*. The parameter *k* of each sample was drawn from a uniform distribution on (0, 60), and the parameter *b* was drawn from a uniform distribution on (0, 600). There was no statistically significant difference between the adjusted sample Gini coefficient (*g*_adj_, calculated by Equation 15) and *G*_*G*_ (calculated by Equation 29), because the 95% CI of *G*_*G*_ versus *g*_adj_ included unity, and the *p*‐value of the paired Wilcoxon signed‐rank test was greater than 0.05 (see Figure 7a). It is clear from Equation (31) that the skewness is equal to 0 when the shape parameter *k* approaches positive infinity. Based on Equation (29), the Gini coefficient was calculated to be equal to 0 at this point as well using the “integrate” function of the “stats” package (Piessens et al. 1983) in R (version 4.3.3; R Core Team 2024), which means that it is feasible to determine whether the data are right‐skewed by calculating whether the Gini coefficient is greater than 0 in the event that the data follow the gamma distribution.

### 5.5 The Gini Coefficient for Distributions With Non‐Zero Thresholds

For linear changes, the Gini coefficient is only affected by the intercept term, irrespective of the slope term. Given two variables *X*, *Y*, and *Y* = *aX* + *c*, where *a* and *c* are constants, the following relationship exists between their Gini coefficients:

(32) $$G_{Y}=\frac{G_{X}}{1+c/\mu }$$

where *μ* is the expected value of *X*. The distributions include the two‐parameter Weibull, two‐parameter lognormal, and one‐parameter exponential, all with a threshold of 0. When the threshold *c* in Equation (32) is unequal to zero (i.e., in the situation of three‐parameter Weibull, three‐parameter lognormal, and two‐parameter exponential distributions), it is equivalent to a linear change in the original distribution with a slope of 1 and an intercept of *c*, and the corresponding Gini coefficients would undergo a transition in Equation (32). However, the statistics like variance and skewness are unaffected by *c*.

**FIGURE 8:**
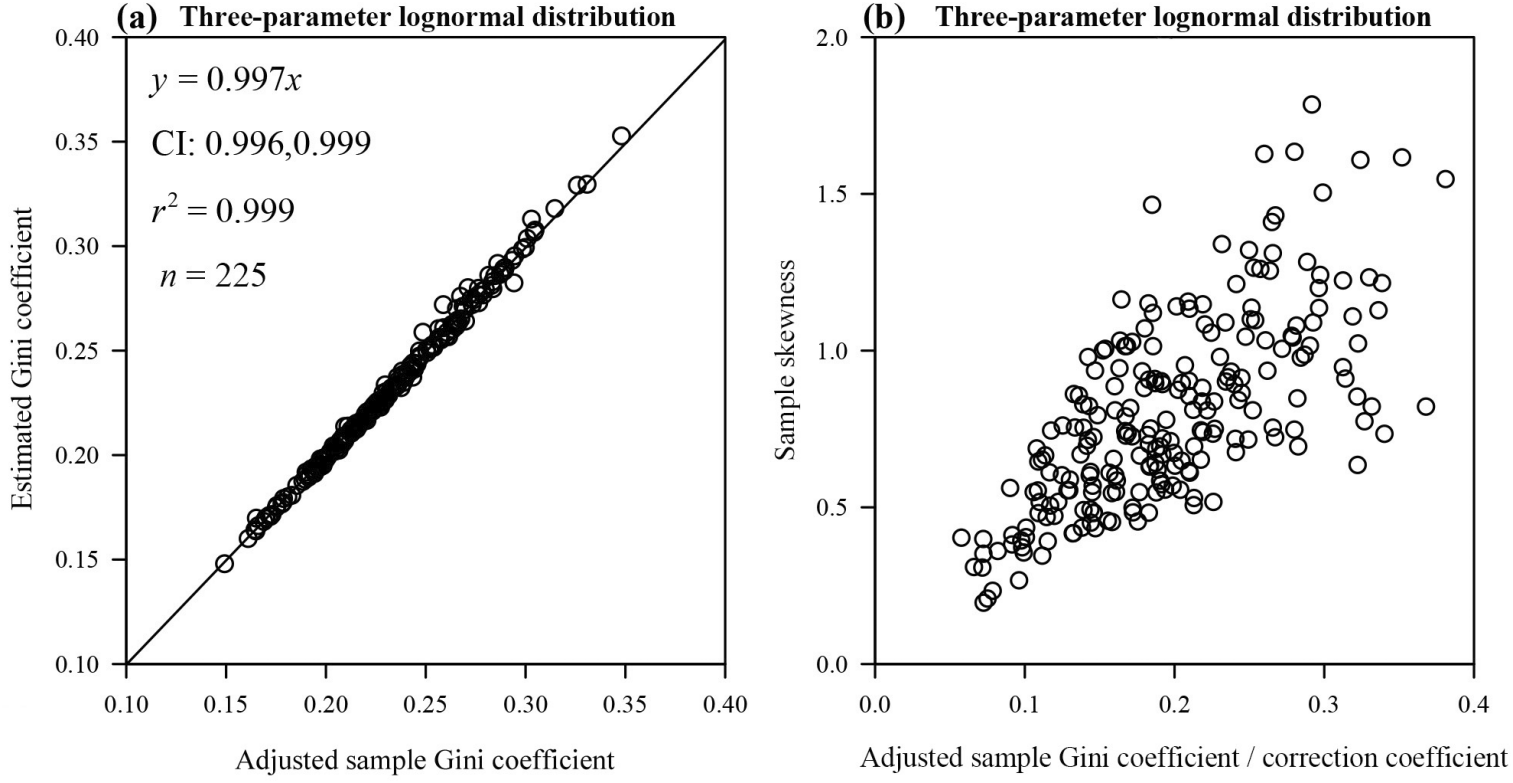
Linear fits to the esimated Gini coefficient vs. the adjusted sample Gini coefficient for 225 sets of the *S. viridis* seedhead length data (a), and scatterplot of adjusted sample Gini coefficient divided by correction coefficient and sample skewness calculated from 225 sets of the *S. viridis* seedhead length data (b). In each panel, the open circles represent the simulated samples, *y* denotes the estimated Gini coefficient calculated by Equations (24) and (32) (a) (or the sample skewness calculated by Equation 12 (b)), and *x* denotes the adjusted sample Gini coefficient (or the adjusted sample Gini coefficient divided by correction coefficient) calculated by Equation (15); *r* ^2^ represents the coefficient of determination; the straight line represents the regression line.

225 data sets of the *S. viridis* seedhead length all passed the three‐parameter lognormal distribution test, and 200 of the 225 data sets passed the two‐parameter lognormal distribution test, indicating that the data conformed to the lognormal distribution and the threshold was not all zero (see Table S4). The parameters of the three‐parameter lognormal distribution for the 225 data sets of *S. viridis* seedhead length data were estimated using the MLE, and the estimated Gini coefficients of the 225 data sets of seedhead length data were further calculated by using Equations (24) and (32). There was no statistically significant difference between the *g*_adj_ (calculated by Equation 15) and the estimated Gini coefficient (calculated by Equations 24 and 32), as the *p*‐value of the paired Wilcoxon signed‐rank test was greater than 0.05 (see Figure 8a).

We back‐corrected the 225 sample Gini coefficients, i.e. divided by the correction coefficient, which resulted in a positive correlation with the sample skewness (Figure 8b), where the correction coefficient employed an estimate, i.e., obtained by taking the parameter obtained from MLE into the correction coefficient's expression.

For uniform, normal, two or three‐parameter Weibull, two or three‐parameter lognormal, one or two‐parameter exponential, gamma distribution, any of these distribution test *p*‐value greater than 0.05 of the data, we can summarize, if the data are symmetric, the skewness value would be small close to 0, at this time the Gini coefficient approximately ranged from 0.56 to 0.58 multiplied by the standard deviation divided by the mean (0.56–0.58 times coefficient of variation) (Equations 19 and 20). In the asymmetric case, if the data conform to the distribution whose threshold is equal to 0, the Gini coefficient can be interpreted as a monotonic‐increasing function of skewness; at this time, the two coefficients are equivalent; both can be recommended. Suppose the threshold value is not equal to 0. In that case, the Gini coefficient could be corrected on the influence of the skewness and the correction coefficient is $1/\left(1+c/\mu \right)$.

## 6 Discussion

### 6.1 How to Use the Distribution‐Based Gini Coefficients?

In this section, we exhibited examples of how to use the above Gini coefficients in an ecological context. For example, Fernandez‐Tschieder and Binkley (2018) refute the use of the Gini coefficient at a time point to assess the degree of competition by using a set of hypothetical stands and calculating the change in the corresponding Gini coefficients after these stands experience three different types of competition for resources (asymmetric, symmetric, and inverse asymmetric competition) per unit of time. Here, an exponential relationship between tree growth and tree size was used for asymmetric competition, a linear relationship between tree growth and tree size was used for both symmetric competition and inverse asymmetric competition, with a zero intercept for symmetric competition and a positive intercept for inverse asymmetric competition. They found that the Gini coefficients become larger after experiencing asymmetric competition, unchanged after symmetric competition, and smaller after experiencing the inverse asymmetric competition, so that it's possible that different stands have the same Gini coefficient after experiencing different types of competition (Fernandez‐Tschieder and Binkley 2018). The reason for this change in the Gini coefficient actually results from the fact that symmetric competition and inverse asymmetric competition can be reflected by a linear size‐growth relationship, with a zero intercept and a positive intercept, and the original stands data plus growth per unit of time becomes the current stand, while the Gini coefficient is not affected by the slope under linear change but only by the intercept, which decreases with the intercept increasing, so the Gini coefficient of the symmetric competition would be unchanged, and the Gini coefficient for inverse asymmetric competition becomes smaller.

In the past few years, there has been a growing interest in tree size diversity in boreal forests, largely due to increasing concerns about the management of uneven‐aged forests, biodiversity, and social values (Lexerød and Eid 2006). To develop management plans that are sustainable in terms of ecological and economic values, indicators that can objectively measure tree size diversity are needed (Lexerød and Eid 2006). Lexerød and Eid (2006) evaluated different indices describing the diversity of diameters using 16 simulated diameter distributions (J‐inverted shaped, normal, and uniform distributions with different parameters) and 174 empirical diameter distributions. The Gini coefficient was found to have the ability to distinguish different distributions (being the only one whose index value is lower under the normal distribution than under the uniform distribution, compared to Simpson's, Berger‐Parker's, and Shannon's indices that have difficulty in distinguishing between the uniform and normal distributions). In fact, the mathematical expressions for the Gini coefficient under the uniform and normal distributions show that the numerical value of the Gini coefficient under the uniform distribution is greater than that under the normal distribution on the condition that the mean and variance are the same.

Huang et al. (2023) discussed the relationships between inequality measures such as the Gini coefficient, coefficient of variation, Theil index, and mean log deviation index using the empirical leaf area data of 240 individual shoots of *Shibataea chinensis* Nakai, and found that the Gini coefficient and the coefficient of variation were linearly and positively correlated. This results from that fact that leaf area data of *S. chinensis* follow the two‐parameter Weibull distributions, and the shape parameters were estimated to range from 2.45 to 15.18. The Gini coefficient for the two‐parameter Weibull distribution is a mono‐increasing function of the shape parameter, and the coefficient of variation for the two‐parameter Weibull distribution $\left(\mathrm{CV}=\sqrt{\frac{2\Gamma \left(2/\beta \right)}{\Gamma \left(1/\beta \right)}-1}\right)$ is predicted to be greater than 0 when the shape parameter ranges from 2 to 16.

In addition, Bendel et al. (1989) argued that under the three‐parameter log‐normal distribution, the Gini coefficients may or may not be correlated with the skewness and can only conservatively recommend skewness independent of threshold, whereas now their relationship can be explicitly provided through their expressions.

The Gini coefficient captures more information than skewness, but this brings benefits as well as drawbacks to the Gini coefficient. The Gini coefficient is subject to mean and threshold values, which somewhat diminishes its ability to show inequality. Péron (2023) found that this is well compensated for by Hill's tail index, which is computed on small samples, usually less than 10% of the total sample size, and more focused on the tail behavior of the distribution. This means that the Gini coefficient and the Hill's tail index used together can compensate for each other and perhaps bring a different spark.

### 6.2 Generalizability of Adjustment Methods Based on Small Sample Sizes

Various methods exist for calculating the sample Gini coefficient. One common approach is to compute the area of a polygon (or set of trapezoids), as utilized in this study. Another method involves specifying a particular functional form of the Lorenz curve and estimating it using nonlinear least squares, then calculating the Gini coefficient by integrating the estimated Lorenz function. Previous studies have shown that sample size‐based adjustment is highly effective for sample Gini coefficients calculated from the area of polygons (trapezoidal sets) (Deltas 2003).

We investigated whether this adjustment is equally effective for sample Gini coefficients calculated by fitting a nonlinear Lorenz curve. The method proposed by Lian et al. (2023) was selected for its high accuracy in fitting the Lorenz function to calculate the Gini coefficient. This method uses the performance equation to fit coordinate‐transformed Lorenz curves, which are then rotated 135° anticlockwise around the origin and shifted to the right by $\sqrt{2}$ (Figure 4c,d). Subsequently, the Gini coefficient is calculated based on the fitted Lorenz curve after rotational translation (Huey and Stevenson 1979; Lian et al. 2023). We assessed whether this adjustment effectively improves the accuracy of the calculation of the sample Gini coefficient.

**FIGURE 9:**
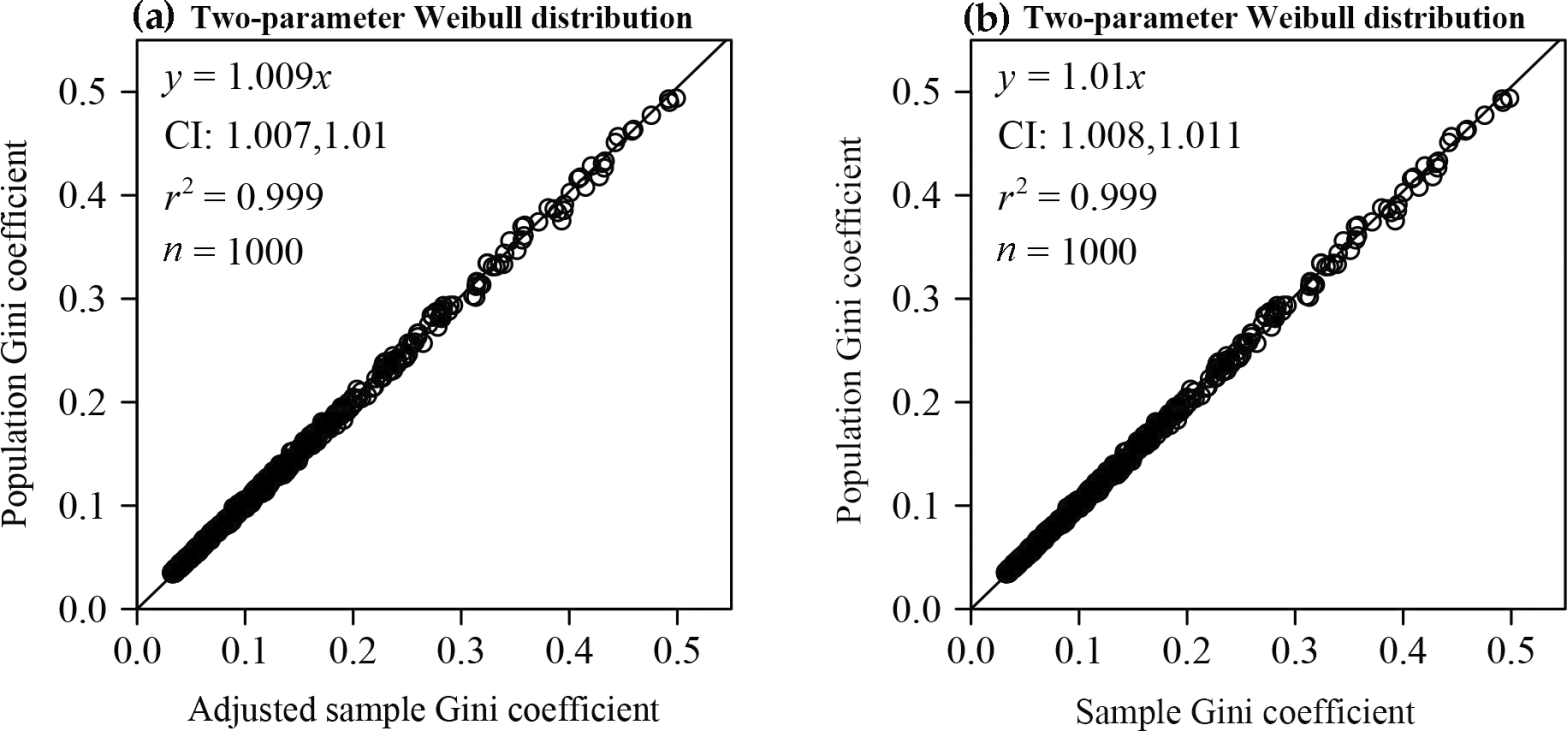
Linear fits to the population Gini coefficient vs. the (adjusted) sample Gini coefficient for 1000 simulated samples from the two‐parameter Weibull distribution. Here, *y* denotes the population Gini coefficient calculated by Equation (16), and *x* denotes the adjusted sample Gini coefficient calculated by the rotated Lorentz method and adjusted for sample size (Equation 15) (a) or sample Gini coefficient calculated by the rotated Lorentz curve method not adjusted for sample size (b). CI represents the 95% confidence interval of the slope; *r* ^2^ represents the coefficient of determination; the straight line represents the regression line.

We conducted 1000 random samplings with 1000 random numbers for each sampling from the two‐parameter Weibull distribution defined by the shape parameter *β* and the fixed scale parameter *α* = 1. The shape parameter *β* of each sample was drawn from a uniform distribution on (1, 20). Cases where the distribution is extremely right‐skewed (the density value steadily decreases) were excluded (*β <* 1), as this approach does not perform well when the size distribution is extremely right‐skewed (Shi, Deng, and Niklas 2024). Figure 9 demonstrates that the adjustment based on a small sample size improves the accuracy of the Gini coefficient calculated by rotating the Lorenz curve, but it does not improve to the same extent as the Gini coefficient calculated by the polygonal (trapezoidal sets) area method.

## 7 Conclusions

This study provided the mathematical expression between the Gini coefficient and skewness for the two‐parameter Weibull distribution. The present study proposed a method to calculate the Gini coefficient under the two‐parameter Weibull distribution by estimating the shape parameter value from samples using the maximum likelihood method and substituting it into the Gini coefficient expression (which is a univariate function of the shape parameter under the two‐parameter Weibull distribution). In the Discussion section, the Gini coefficient–skewness relationships for other common biological size distributions were also analyzed, including the uniform, normal, lognormal, gamma, three‐parameter Weibull, three‐parameter lognormal, and three‐parameter gamma distributions. The mathematical expressions of the Gini coefficients for these distributions were provided as well and their relationships with skewness were analyzed, which can be divided into three types: (i) for a symmetric distribution, the skewness is 0, the Gini coefficient ranges from 0.56 to 0.58 multiplied by the standard deviation divided by the mean irrespective of the skewness; (ii) for an asymmetric distribution with a zero threshold, the Gini coefficient is a monotonically increasing function of the skewness and both are equal to whichever one can be selected; and (iii) for an asymmetric distribution with a non‐zero threshold, the Gini coefficient is affected by the skewness and a correction factor as well.

The approach was validated using both simulation data and empirical data (tree diameter at breast height data in a forest census, and seedhead length data of *S. viridis* ). The study investigated whether this small‐sample‐based adjustment of the Gini coefficient improved computational accuracy equally for other methods of calculating the Gini coefficient. The results show that the small sample‐based adjustment was still useful for the rotated Lorenz curve method (one of the most accurate methods for calculating the Gini coefficient using the Lorenz curve) but not as effective as the trapezoidal area method. In conclusion, this study enhances understanding of the relationship between the Gini coefficient and the skewness coefficient, aids in selecting and using the coefficient for calculating the degree of inequality, and broadens the methods for calculating the Gini coefficient.

## Author Contributions

**Meng Lian:** conceptualization (lead), formal analysis (lead), methodology (lead), writing – original draft (equal). **Long Chen:** writing – review and editing (equal). **Cang Hui:** writing – original draft (equal). **Fuyuan Zhu:** supervision (equal), writing – review and editing (equal). **Peijian Shi:** supervision (equal), writing – review and editing (equal).

## Conflicts of Interest

The authors declare no conflicts of interest.

## Supporting information


**Table S1.** Tree diameter at breast height data for each quadrat.
**Table S2**. Estimated parameters of the Weibull distribution function and inequality measures for each of the 160 quadrats of tree diameter at breast height data.
**Table S3**. Seedhead length data of *Setaria viridis* (L.) P. Beauv.
**Table S4**. Calculation results for 225 quadrats of seedhead length data of *Setaria viridis* (L.) P. Beauv.

## Data Availability

The forest survey data are accessible in dryad, an online repository, at https://doi.org/10.5061/dryad.h9w0vt4np.

## References

[ece370637-bib-0001] Baddeley, A. , E. Rubak , and R. Turner . 2015. Spatial Point Patterns: Methodology and Applications With R. New York: CRC Press.

[ece370637-bib-0002] Bendel, R. B. , S. Higgins , J. E. Teberg , and D. Pyke . 1989. “Comparison of Skewness Coefficient, Coefficient of Variation, and Gini Coefficient as Inequality Measures Within Populations.” Oecologia 78: 394–400. 10.1007/BF00379115. 28312587

[ece370637-bib-0003] Bolker, B. 2023. “bbmle: Tools for General Maximum Likelihood Estimation.” R Package Version 1.0.25.1. Accessed January 1, 2024. https://CRAN.R‐project.org/package=bbmle.

[ece370637-bib-0004] Brent, R. 1973. Algorithms for Minimization Without Derivatives. Englewood Cliffs, NJ: Prentice‐Hall.

[ece370637-bib-0005] Crow, E. 1988. Lognormal Distributions: Theory and Applications. New York: Routledge.

[ece370637-bib-0006] Deltas, G. 2003. “The Small‐Sample Bias of the Gini Coefficient: Results and Implications for Empirical Research.” Review of Economics and Statistics 85: 226–234. 10.2139/ssrn.224896.

[ece370637-bib-0007] Diaz, R. M. , and S. K. M. Ernest . 2024. “Temporal Changes in the Individual Size Distribution Modulate the Long‐Term Trends of Biomass and Energy Use of North American Breeding Bird Communities.” Global Ecology and Biogeography 33: 74–84. 10.1111/geb.13777.

[ece370637-bib-0008] Dillon, K. T. , A. N. Henderson , A. G. Lodge , et al. 2019. “On the Relationships Between Size and Abundance in Plants: Beyond Forest Communities.” Ecosphere 10: e02856. 10.1002/ecs2.2856.

[ece370637-bib-0009] Fernandez‐Tschieder, E. , and D. Binkley . 2018. “Linking Competition With Growth Dominance and Production Ecology.” Forest Ecology and Management 414: 99–107. 10.1016/j.foreco.2018.01.052.

[ece370637-bib-0010] Gilbert, P. , and R. Varadhan . 2019. “numDeriv: Accurate Numerical Derivatives.” R Package Version 2016.8‐1.1. Accessed January 1, 2024. https://CRAN.R‐project.org/package=numDeriv.

[ece370637-bib-0011] Gini, C. 1912. Variability and Mutability: Contribution to the Study of Distributions and Statistical Relationships. Bologna, Italy: Paolo Cuppini Typography.

[ece370637-bib-0012] Hara, T. 1988. “Dynamics of Size Structure in Plant Populations.” Trends in Ecology & Evolution 3: 129–133. 10.1016/0169-5347(88)90175-9. 21227181

[ece370637-bib-0041] Huang, W. , K. Ma , and D. K. Gladish . 2024. “Ellipse or Superellipse for Tree‐ring Geometries? Evidence from Six Conifer Species.” Trees 38: 1403–1413. 10.1007/s00468-024-02561-2.

[ece370637-bib-0013] Huang, L. C. , D. A. Ratkowsky , C. Hui , et al. 2023. “Inequality Measure of Leaf Area Distribution for a Drought‐Tolerant Landscape Plant.” Plants 12: 3143. 10.3390/plants12173143. 37687388 PMC10490070

[ece370637-bib-0014] Huey, R. B. , and R. D. Stevenson . 1979. “Integrating Thermal Physiology and Ecology of Ectotherms: A Discussion of Approaches.” American Zoologist 19: 357–366. 10.1093/icb/19.1.357.

[ece370637-bib-0015] Jambunathan, M. V. 1954. “Some Properties of Beta and Gamma Distributions.” Annals of Mathematical Statistics 25: 401–405.

[ece370637-bib-0016] Joanes, D. N. , and C. A. Gill . 1998. “Comparing Measures of Sample Skewness and Kurtosis.” Journal of the Royal Statistical Society Series D: The Statistician 47: 183–189.

[ece370637-bib-0017] Kokko, H. , A. Mackenzie , J. D. Reynolds , J. Lindström , and W. J. Sutherland . 1999. “Measures of Inequality Are Not Equal.” American Naturalist 154: 358–382. 10.1086/303235. 10506550

[ece370637-bib-0018] Lai, C. D. 2014. Generalized Weibull Distributions. SpringerBriefs in Statistics. Berlin, Heidelberg: Springer.

[ece370637-bib-0019] Lai, C. D. , D. N. P. Murthy , and M. Xie . 2011. “Weibull Distributions.” WIREs Computational Statistics 3: 282–287. 10.1002/wics.157.

[ece370637-bib-0020] Lexerød, N. , and T. Eid . 2006. “An Evaluation of Different Diameter Diversity Indices Based on Criteria Related to Forest Management Planning.” Forest Ecology and Management 222: 17–28. 10.1016/j.foreco.2005.10.046.

[ece370637-bib-0021] Lian, M. , P. J. Shi , L. Y. Zhang , W. H. Yao , J. Gielis , and K. J. Niklas . 2023. “A Generalized Performance Equation and Its Application in Measuring the Gini Index of Leaf Size Inequality.” Trees 37: 1555–1565. 10.1007/s00468-023-02448-8.

[ece370637-bib-0022] Lopes, R. H. C. 2011. “Kolmogorov‐Smirnov Test.” In International Encyclopedia of Statistical Science, edited by M. Lovric , 718–720. Berlin: Springer.

[ece370637-bib-0023] Lorenz, M. O. 1905. “Methods of Measuring the Concentration of Wealth.” American Statistical Association 9: 209–219. 10.2307/2276207.

[ece370637-bib-0024] Mateu‐Figueras, M. G. , and R. A. Olea . 2022. “Lognormal Distribution.” In Encyclopedia of Mathematical Geosciences. Encyclopedia of Earth Sciences Series, edited by B. Daya Sagar , Q. Cheng , J. McKinley , and F. Agterberg , 1–4. Cham: Springer.

[ece370637-bib-0025] Péron, G. 2023. “Reproductive Skews of Territorial Species in Heterogeneous Landscapes.” Oikos 2: e09627. 10.1111/oik.09627.

[ece370637-bib-0026] Piessens, R. , E. Doncker‐Kapenga , C. Uberhuber , and D. Kahaner . 1983. Quadpack: A Subroutine Package for Automatic Integration. Berlin, Heidelberg: Springer.

[ece370637-bib-0027] R Core Team . 2024. R: A Language and Environment for Statistical Computing. Vienna: R Foundation for Statistical Computing. https://www.R‐project.org/.

[ece370637-bib-0028] Sarabia, J. M. 1997. “A Hierarchy of Lorenz Curves Based on the Generalized Tukey's Lambda Distribution.” Econometric Reviews 16: 305–320. 10.1080/07474939708800389.

[ece370637-bib-0029] Shapiro, S. S. , and M. B. Wilk . 1965. “An Analysis of Variance Test for Normality (Complete Samples).” Biometrika 52: 591–611. 10.1093/BIOMET/52.3-4.591.

[ece370637-bib-0030] Shi, P. J. , L. L. Deng , and K. J. Niklas . 2024. “Rotated Lorenz Curves of Biological Size Distributions Follow Two Performance Equations.” Symmetry 16: 565. 10.3390/sym16050565.

[ece370637-bib-0031] Shi, P. J. , J. Gao , Z. P. Song , Y. H. Liu , and C. Hui . 2018. “Spatial Segregation Facilitates the Coexistence of Tree Species in Temperate Forests.” Forests 9: 768. 10.3390/f9120768.

[ece370637-bib-0032] Shi, P. J. , B. K. Quinn , L. Chen , J. Gao , and J. Schrader . 2023. “Forest Survey Data of a Landscape in Pine Mountain, Dryad, Dataset.” Accessed October 4, 2023. 10.5061/dryad.h9w0vt4np.

[ece370637-bib-0033] Solbrig, O. T. 1981. “Studies on the Population Biology of the Genus *Viola* II. The Effect of Plant Size on Fitness in *Viola sororia* .” Evolution 35: 1080–1093. 10.1111/j.1558-5646.1981.tb04977.x. 28563389

[ece370637-bib-0034] Student [W. S. Gosset] . 1908. “The Probable Error of a Mean.” Biometrika 6: 1–25. 10.2307/2331554.

[ece370637-bib-0035] Taubert, F. , F. Hartig , H. J. Dobner , and A. Huth . 2013. “On the Challenge of Fitting Tree Size Distributions in Ecology.” PLoS One 8: e58036. 10.1371/journal.pone.0058036. 23469137 PMC3585190

[ece370637-bib-0036] Teimouri, M. , K. Abdolahnezhad , and S. Ghalandarayeshi . 2020. “Evaluation of Estimation Methods for Parameters of the Probability Functions in Tree Diameter Distribution Modeling.” Environmental Resources Research 8: 25–40. 10.22069/ijerr.2020.5086.

[ece370637-bib-0037] Weibull, W. 1951. “A Statistical Distribution Function of Wide Applicability.” Journal of Applied Mechanics 18: 293–297. 10.1115/1.4010337.

[ece370637-bib-0038] Weiner, J. , and O. T. Solbrig . 1984. “The Meaning and Measurement of Size Hierarchies in Plant Populations.” Oecologia 61: 334–336. 28311058 10.1007/BF00379630

[ece370637-bib-0039] Weiner, J. , and S. C. Thomas . 1986. “Size Variability and Competition in Plant Monocultures.” Oikos 47: 211–222. 10.2307/3566048.

[ece370637-bib-0040] Wilcoxon, F. 1945. “Individual Comparisons by Ranking Methods.” Biometrics Bulletin 1: 80–83. 10.2307/3001968.

